# A tool for calculating binding-site residues on proteins from PDB structures

**DOI:** 10.1186/1472-6807-9-52

**Published:** 2009-08-03

**Authors:** Jing Hu, Changhui Yan

**Affiliations:** 1 Department of Computer Science, Utah State University, Logan, UT, USA; 2 Department of Mathematics & Computer Science, Franklin & Marshall College, Lancaster, PA, USA

## Abstract

**Background:**

In the research on protein functional sites, researchers often need to identify binding-site residues on a protein. A commonly used strategy is to find a complex structure from the Protein Data Bank (PDB) that consists of the protein of interest and its interacting partner(s) and calculate binding-site residues based on the complex structure. However, since a protein may participate in multiple interactions, the binding-site residues calculated based on one complex structure usually do not reveal all binding sites on a protein. Thus, this requires researchers to find all PDB complexes that contain the protein of interest and combine the binding-site information gleaned from them. This process is very time-consuming. Especially, combing binding-site information obtained from different PDB structures requires tedious work to align protein sequences. The process becomes overwhelmingly difficult when researchers have a large set of proteins to analyze, which is usually the case in practice.

**Results:**

In this study, we have developed a tool for calculating binding-site residues on proteins, TCBRP . For an input protein, TCBRP can quickly find all binding-site residues on the protein by automatically combining the information obtained from all PDB structures that consist of the protein of interest. Additionally, TCBRP presents the binding-site residues in different categories according to the interaction type. TCBRP also allows researchers to set the definition of binding-site residues.

**Conclusion:**

The developed tool is very useful for the research on protein binding site analysis and prediction.

## Background

Proteins perform various functions through interactions with other molecules, such as DNA, RNA, proteins, carbohydrates, and ligands. To understand the mechanisms of these interactions, many studies have been performed to analyze the properties of binding sites on proteins, such as residue composition, secondary structure, geometric shape, electrostatic potentials, etc [1-10]. To perform such an analysis, researchers must first identify the amino acid residues (referred to as *binding-site residues*) that are involved in the interactions. In other studies where the goal is to build computational predictors for predicting functional sites on proteins (e.g. DNA-binding sites, RNA-binding sites, protein-protein binding sites), researchers also need to identify binding-site residues on the training and test sets to train and evaluate their predictors [11-17].

**Figure 1:**
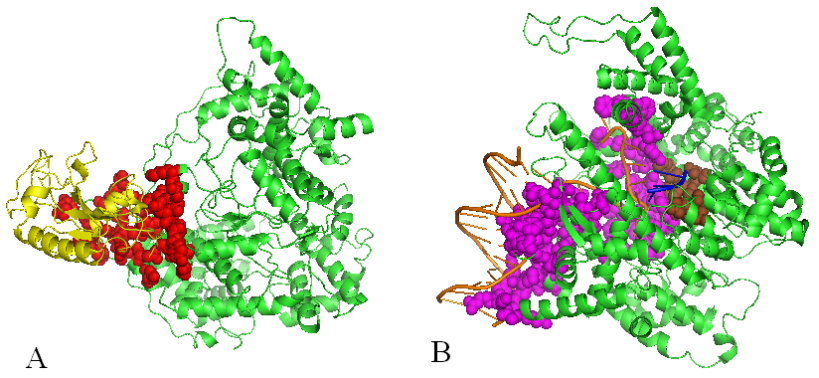
**Binding-site residues on the T7 RNA polymerase shown by different PDB structures**. **A**: PDB id 1ARO: A complex of T7 RNA polymerase with T7 Lysozyme. Green: T7 RNA polymerase; Yellow: T7 Lysozyme; Red: protein-binding residues on T7 RNA polymerase; **B**: PDB id 1QLN: A complex of T7 RNA polymerase with DNA and RNA. Green: T7 RNA polymerase; Orange: DNA; Blue: RNA; Magentas: DNA-binding residues; Brown: RNA-binding residues.

**Figure 2:**
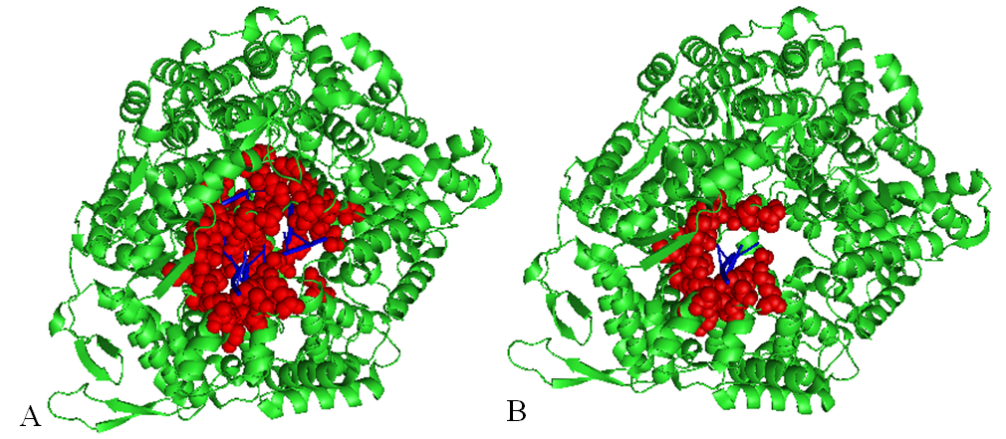
**Different PDB structures show different RNA-binding residues on the retrovirus polymerase lambda-3**. **A**: PDB id 1UON; **B **PDB id 1N1H. Blue: RNA; Green: retrovirus polymerase lambda-3; Red: RNA-binding residues on lambda-3.

In most, if not all, of the above-mentioned studies, the binding-site residues are calculated from the 3-dimensional (3D) structures deposited in Protein Data Bank (PDB) [18]. Usually, researchers collected a non-redundant set of PDB structures, and then calculated binding-sites based on the PDB structures. However, one serious problem with this approach is that a protein may have multiple binding sites that interact with different interacting partners, but one PDB structure usually does not show all of these interactions. For example, T7 RNA polymerase appears in both PDB 1ARO and 1QLN. 1ARO reveals the binding-site residues on T7 RNA polymerase that are involved in the protein-protein interaction (red color in Figure 1A), while 1QLN reveals the binding-site residues on T7 RNA polymerase that are involved in DNA binding (magentas color in Figure 1B) and RNA binding (brown color in Figure 1B). Even when two PDB structures reveal the same type of interaction on the same protein, the binding-sites can still be different depending on the interacting partner. For example, both 1UON and 1N1H are a complex of retrovirus polymerase lambda-3 with RNA, but 1UON shows that the RNA-binding site on lambda-3 consists of 59 residues (red color in Figure 2A), while 1N1H shows that the RNA-binding site of lambda-3 contains only 27 residues (red color in Figure 2B).

Thus, for the same protein, different sets of binding-site residues might be obtained depending on the PDB structure that is considered, and a residue of a protein may be defined as binding-site residue in one PDB structure but as non-binding-site residue in another. This inconsistency can cause serious problems in research. Thus, for a given protein, researchers need to identify all PDB structures that contain the protein, and calculate binding-site residues on the protein using all of them.

After users have found all the PDB structures that contain a given protein, the protein sequences shown in different PDB structures must be aligned properly to combine the binding-site information obtained from different structures. This step is not as simple as it may first appear. It cannot be done by matching the sequence indexes of residues in the PDB structures, because the same protein chain may have different sequence indexing in different PDB structures. For example, 1qqi_A and 1gxp_A are the same protein chain in different PDB structures. In PDB 1gxp, the first residue in chain A is ALA with sequence index of 127. However, the same residue in PDB 1qqi has an index of 2. It can neither be done by performing a simple one-to-one mapping between the two sequences from head to tail, because residue missing occurs frequently in PDB structures. Thus, researchers need a tool that can efficiently combine binding-site information from different PDB structures.

The abovementioned needs become overwhelmingly impressive when users have a large set of proteins to analyze. Against these needs, we have developed TCBRP, a tool for calculating binding-site residues on proteins. For an input protein, TCBRP can quickly find all binding-site residues on it by integrating binding-site information obtained from all PDB structures that contain the protein of interest. Additionally, the TCBRP presents the binding-site residues by categories based on the type of the molecule that they contact, e.g. DNA, RNA, protein, carbohydrates, and ligands. An extra benefit of TCBRP is that it allows users to choose the definition of binding-site residues.

## Implementation

**Figure 3:**
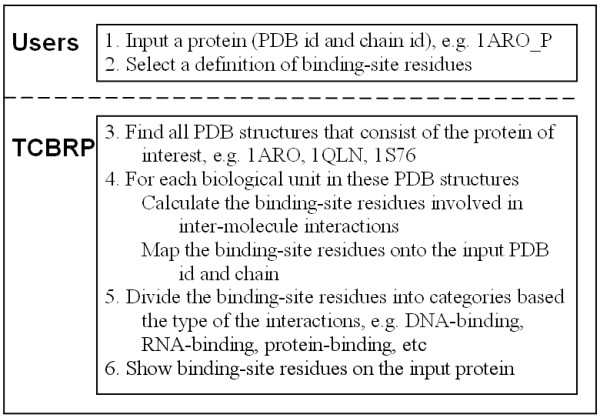
**The schema of TCBRP**.

**Figure 4:**
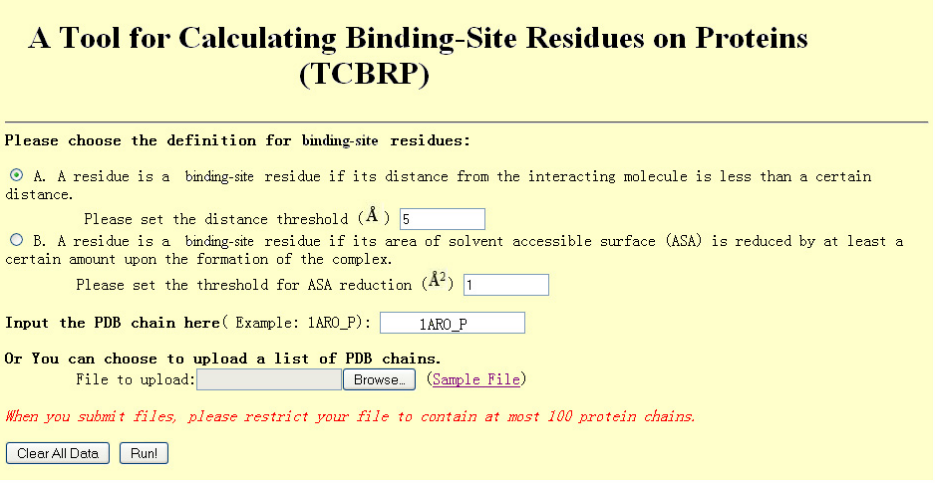
**Input form of TCBRP**.

Figure 3 shows the schema of TCBRP. First, users input a protein of interest and choose a definition of binding-site residues. There are two types of definition for binding-site residues. One is based on the reduction of solvent accessible surface upon the formation of complex [7]. A residue is defined to be a binding-site residue if its solvent accessible surface area (ASA) is reduced by at least a certain amount (default threshold is 1 Å^2^) during the formation of the complex. The second definition is based on the atom distance [5]. A residue is defined as a binding-site residue if its distance to the interacting partner is less than a certain distance (default threshold is 5 Å). For both definitions, users can set the threshold (See figure 4).

Upon the input, TCBRP searches the entire PDB database to find all the complex structures that contain a protein that shares at least 95% sequence similarity with the input protein. Then, the biological units derived from these structures are used to calculate binding-site residues on the protein of interest. We use biological units instead of the raw PDB structure because the biological units show the functional state of the protein in life systems. Additionally, using biological units can avoid the artificial interactions that due to artificial packing in the raw PDB structures. TCBRP focuses on the binding sites involved in inter-molecule interactions, because they correspond to functional sites on proteins. Intra-molecule interactions that involve residues from the same chain or from different chains of the same molecule are discarded.

To reveal all binding sites on the input protein, the binding-site residues obtained from different biological units must be mapped to the input protein. To do this, the sequences of the different copies of the protein in different PDB structures must be aligned properly. In TCBRP, this step is achieved by aligning the protein sequences in PDB structures with the protein sequence found in the Uniprot [19] using global alignment.

**Figure 5:**
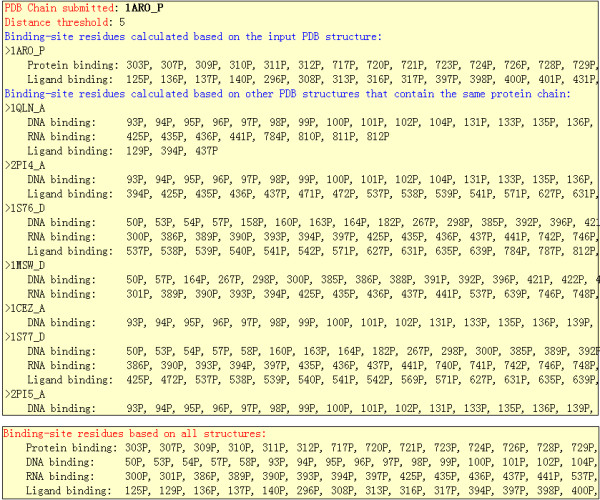
**The return form of TCBRP**. The upper box shows the binding-site residues mapped on the input protein, 1ARO_P, when different PDB structures are used to calculate binding sites. The lower box shows all the binding-site residues on the input protein by combing all the results.

Proteins are involved in various functions. Depending on the interacting partner, the binding sites on proteins can be divided into different categories, such as DNA-binding sites, RNA-binding sites, protein-protein binding sites, carbohydrate-binding sites, and ligand-binding sites. In many studies, researchers like to distinguish different types of protein binding sites. In response to this need, when a protein is involved in different types of interactions, the TCBRP show the binding-site residues for every type of interaction separately (Figure 5).

Using TCBRP, users can input one protein chain in a time, or input a file of protein chains in a batch. For a protein that consists of multiple chains, users can submit a file consisting of all the chains, then the TCBRP will show the binding-site residues on each chain that are involved in inter-molecule interactions.

## Results and discussion

Figures 4 and 5 show an example of input and output for TCBRP. Assume that 1ARO is one of the PDB structures from which a user wants to find binding sites. Note that 1ARO is a complex of T7 RNA Polymerase (chain P) with T7 Lysozyme (Chain I). Without TCBRP, the user may use 1ARO to calculate binding-site residues on T7 RNA polymerase and only find 26 binding-site residues that correspond to the interaction between T7 RNA Polymerase and T7 Lysozyme. However, RNA polymerase interacts with multiple molecules including RNA, DNA, and proteins. In the research on functional site prediction and analysis, the user will need to find all the functional sites on the T7 RNA polymerase. To obtain these results without TCBRP, the user would need to go through a long and painful process of finding all complexes that contain T7 RNA polymerase, calculating binding-site residues using each of the complexes, and combining the information given by different structures. Using TCBRP, the user can obtain the results easily. Figure 4 shows the input page. Here, the input is the P chain of 1ARO, which is T7 RNA polymerase. Upon the input, the TCBRP automatically finds all PDB structures that contain T7 RNA polymerase, i.e. 1ARO, 1QLN, 2PI4, 1S76, 1MSW, 1CEZ, 1S77, and 2PI5. Binding-sites on T7 RNA polymerase are then calculated based on each of these structures and mapped to the sequence of the input chain 1ARO_P (upper box in figure 5). In the end, the TCBRP combines all the results and shows the binding-site residues involved in each type of interaction separately (lower box in figure 5), which include 26 residues in protein-protein binding sites, 112 in DNA-binding, 28 RNA-binding sites, and 54 ligand-binding residues.

## Conclusion

Many studies have been conducted on protein functional site prediction and analysis. Calculating binding-site residues on proteins based on the PDB structures has been a necessary and yet painful and time-costly step for these studies. TCBRP has been developed to address this problem with ease. Using TCBRP, users will be able to collect all binding-site residues on proteins of interest very quickly. The developed web server will be useful for the studies on protein interaction and protein functional sites.

## Availability and requirements

• **Project name**: A tool for calculating binding-site residues on proteins from PDB structures

• **Project home page**: 

• **Operating system(s)**: Platform independent

## Authors' contributions

CY conceived of the project, designed the architecture, supervised the implementation, and drafted and revised the manuscript. JH performed the coding and attended the discussions. All authors read and approved the final manuscript.
